# Annotations

**Published:** 1889-10-19

**Authors:** 


### Annotations.

**"In the Vulgar Tongue."** The Prayer-book, which has some claim to the respect even of those who do not use it, made provision centuries ago for the religious instruction of children and others by means of the "vulgar tongue." That was in olden times, when there was one tongue for the "vulgar" and one for the "learned." All the world uses the "vulgar tongue" now, and all knowledge has become the common property of all men who choose to take the trouble to work for it. There still survives here and there, even among educated men, a few antiquated fossils who "dodder" up and down, shaking their impotent umbrellas, and trying to proclaim with squeaking voice the impending ruin of the world, because natural knowledge, like theology and metaphysics, is no longer under professional lock and key. If the croakers were young and robust, it might be desirable to take them metaphorically by the collar, and to try to shake a little sense into them or out of them: though we have it on high authority that it is never a profitable proceeding to "cast pearls before swine." Being, however, for the most part old, the "mumblers" must be passed by with respectful silence. They live in a new day, and they cannot understand it; that is all. We are glad to see that at St. Thomas's Hospital the dawn of the new time is understood and welcomed. Mr. William Anderson, F.R.C.S., lecturer on anatomy and surgeon to the skin department of the hospital, opened the medical session and reviewed the growth of the surgical art from the sixteenth century to the present time. After showing the progress that had been made, and the causes which had operated to produce the wonderful expansion in knowledge and advancement in practical skill with which we are now familiar, he affirmed that, beyond all these causes, "a novel and unconventional type of surgical literature in the vulgar tongue played an important part in diffusing knowledge and stimulating enquiry." How can it be otherwise? If you shut up the greatest genius in the world in a country village, he is to his local contemporaries no more than a very peculiar and sometimes even a very "crazy" fellow. But when he is once made known to the great world by means of his novel, or his poem, or his play, he is forthwith put on his proper pedestal as a man of genius and an honour to his age. So it must be with every art, or science, or calling of any conceivable kind. Shut it up in a college, in a clique, in a learned profession, and it is looked at askance as a curious kind of mystery and uncertainty, which may be of service to mankind, or may possibly be more of an enemy than a friend. Nothing better illustrates this than the history of medicine. So long as it was a mere doubtful art, divorced from science and scorned of literature, it was the butt of all men's ridicule, from Bacon downwards. The moment it called science to its aid, it began to be honourable and honoured. As, growing bolder with assured knowledge, it began to show itself to the world in its new lineaments and with its new claims to regard, it passed from honour to esteem, and thence to confidence and love. It is now endeavouring to throw off the last shreds of its crude and embryonic life, and to stand forth openly and boldly before the world in its native simplicity. What, indeed, has modern medicine or surgery to be ashamed of that it should hide itself from public study and criticism? Does it traffic any longer with the occult arts? Is it not open, and sceptical even of its own work and progress, beyond anything known to previous experience? The medicine and surgery of the present time, so far from being occult and mysterious, present an object-lesson to all men of the high worth and value of the scientific method associated with the common and the empirical. Medicine, in short, has a mission to the world which is greater in some respects than even its healing work, the mission of making plain to mankind how pure science may work side by side with an honest empiricism, and may be all the better for the association. The world is not, and never can be, exclusively scientific; but a world of experience guided by science, and of science quickened and made living and beautiful by experience. When the laboratory is married to the plough, the golden age will have fairly set in. All literary effort that hastens the advent of such a marriage deserves and secures the approval of every right-minded man.

**Sir Henry Roscoe on Pasteurism.** Sir Henry Roscoe, in his address to the students at the Midland Institute, admitted, what we have over and over again pointed out in this journal, that the main point of the Pasteur treatment consists in this—viz., that, after Pasteur's inoculation of a person bitten by a rabid animal, there is a race between the powerful virus of the rabid dog and the attenuated virus of the inoculator. If the dog's virus acts the more powerfully and promptly, then the patient must become hydrophobic and die; but if the attenuated virus acts with so much more rapidity than the rabid virus as to catch it up, so to speak, and to pass it by, then it will reach vital centres first, and by its exhausting action render them incapable of being dangerously affected by the powerful poison which is following in its wake. This has been our view of the case from the very outset, a view which we have constantly set forth, despite the authority of the great names on the Pasteur side of the question. It now appears to be Sir Henry Roscoe's; is it also the view of those eminent members of the medical profession whose prompt conversion to Pasteurism was so significant a "sign of the times"? If it be, there is yet hope that British medicine will regain its character for critical acumen and steady sense. It is evident to all impartial persons that Pasteurism has not yet passed the "trial" stage. This one circumstance alone, that practically all the facts before us are produced by one man, and that one man a chemist, not a doctor, ought to give us pause. Was it thus that vaccination won its way? Was it thus that chloroform established its reputation? Did quinine, salicin and the antiseptic system of surgery pass through such slight ordeals as this; and were they then accepted by the "leaders" of the medical profession? The thing is absurd. It is quite right and reasonable to be polite to M. Pasteur, to hear all he says, to consider his facts, and to hold his statements and his work as sufficient grounds for the making of a series of experiments in other civilised countries, and on a large scale; but there is as yet no scientific warrant for more than this. Science happily knows nothing whatever of names and eminences. When a man who has attained to distinction by worthy means announces a new scientific discovery, there is a presumption in its favour, but nothing more. Congratulation of a tentative kind may come first, but criticism must surely follow, and the criticism must be impartial and inflexible. We point out once more that ordinarily, of two viruses of the same or a similar genus, the one that is the more intense in character will act both more quickly and more powerfully than the other. This is so commonly the case, that it may be called a "law of viruses." If a feeble virus acts both more quickly and more powerfully than a strong one, we have at least to deal with an anomaly, with an exception; and any scientific man knows that such apparent exceptions ought always to be suspected; and that, in fact, we ought not to rest content until we have discovered the reason of the apparent exception, until, in short, we have removed the exception from its present classification and placed it under its own proper law. Why does the weak virus of Pasteur, which is the same in kind as the strong virus of the dog, though feebler in intensity—why does it act differently from other viruses allied to it in character, if it does act differently? If it be said that we do not know, then, it must be replied, we can never accept Pasteurism as a truly scientific method of treating hydrophobia until we do know. Of course, it may be accepted as a useful "rule of thumb" method if undoubted facts should prove it to be of value, or even to appear of value; but scientific it cannot be until we find by what unknown "law of viruses" this very peculiar aberration is influenced and brought about.

